# The Role of Long Noncoding RNA (lncRNAs) Biomarkers in Renal Cell Carcinoma

**DOI:** 10.3390/ijms24010643

**Published:** 2022-12-30

**Authors:** Jacek Rysz, Tomasz Konecki, Beata Franczyk, Janusz Ławiński, Anna Gluba-Brzózka

**Affiliations:** 1 Department of Nephrology, Hypertension and Family Medicine, Medical University of Lodz, 113 Żeromskiego Street, 90-549 Lodz, Poland; 2 Department of Urology, Medical University of Lodz, 113 Żeromskiego Street, 90-549 Lodz, Poland; 3 Department of Urology, Institute of Medical Sciences, Medical College of Rzeszow University, 35-055 Rzeszow, Poland

**Keywords:** renal cell carcinoma, long, noncoding RNA, tumorigenesis, metastasis

## Abstract

Renal cell carcinoma is one of the common cancers whose incidence and mortality are continuously growing worldwide. Initially, this type of tumour is usually asymptomatic. Due to the lack of reliable diagnostic markers, one-third of ccRCC patients already have distant metastases at the time of diagnosis. This underlines the importance of establishing biomarkers that would enable the prediction of the disease’s course and the risk of metastasis. LncRNA, which modulates genes at the epigenetic, transcriptional, and post-transcriptional levels, appears promising. The actions of lncRNA involve sponging and sequestering target miRNAs, thus affecting numerous biological processes. Studies have confirmed the involvement of RNAs in various diseases, including RCC. In this review, we focused on MALAT1 (a marker of serious pathological changes and a factor in the promotion of tumorigenesis), RCAT1 (tumour promoter in RCC), DUXAP9 (a plausible marker of localized ccRCC), TCL6 (exerting tumour-suppressive effects in renal cancer), LINC00342 (acting as an oncogene), AGAP2 Antisense1 (plausible predictor of RCC progression), DLEU2 (factor promoting tumours growth via the regulation of epithelial-mesenchymal transition), NNT-AS1 (sponge of miR-22 contributing to tumour progression), LINC00460 (favouring ccRCC development and progression) and Lnc-LSG1 (a factor that may stimulate ccRCC metastasis).

## 1. Introduction

Renal cell carcinoma (RCC) is the seventh most common cancer globally and incidence and mortality are constantly growing worldwide, especially in Western countries [1,2]. There are more than ten subtypes of RCC (clear cell RCC, papillary RCC (pRCC), and chromophobe RCC (chRCC)), of which clear cell renal cell carcinoma (ccRCC) is the most prevalent, accounting for 85–90% of cases [3]. Clear cell renal cell carcinoma is malignant and the rate of metastasis and recurrence following nephrectomy is high (approximately 30% of patients) [4]. The ccRCC course is usually asymptomatic, and no reliable early diagnostic markers are available. Thus, approximately 30% of ccRCC patients have distant metastasis at the time of their initial diagnosis [5]. Based on transcriptomics, patients with ccRCC have been classified into ccA and ccB subtypes, the latter being associated with significantly better disease-specific survival, compared with ccA [6,7,8]. Renal clear cell carcinoma was found to be associated with immune cell infiltration [9]. The estimated five-year overall survival rate for patients with localized disease is 93%, however, the rate of five-year survival in cases of patients with metastatic ccRCC amounts to 12% [10]. The assessment of ccRCC prognosis is based on the AJCC staging system as the gold standard [11]. In turn, the Leibovich score is commonly utilized for the prediction of ccRCC recurrence [12]. This scoring algorithm is based on tumour stage and size, the status of regional lymph node, tumour nuclear grade, as well as histologic tumour necrosis [12]. However, it is not a perfect tool since it properly predicts a five-year metastasis-free survival rate in 97% of low-risk cases. The gold standard for the treatment of RCC involves nephrectomy and chemotherapy [13]. Despite the advancement in treatment methods (targeted therapy and immunotherapy), the five-year overall survival rate in patients with metastatic ccRCC has not improved [14]. Therefore, new therapies are constantly searched for. It appears that treatment based on the inhibition of vital regulatory targets for metastasis may prove the most efficient therapy for patients who have lost the chance for surgery [15]. LncRNAs have been suggested to be the new factors that regulate mechanisms associated with tumour metastasis [16]. A better understanding of molecular and histological heterogeneities of cancers could translate into improved outcomes for patients. There is also a need for new biomarkers which enable the prediction of the prognosis of patients with ccRCC. Due to the fact that the effectiveness of treatment is better when the disease or recurrence is quickly found, there is still a search for sensitive and specific biomarkers which would enable the prediction of the risk of progression, metastasis, etc. [17]. The risk of recurrence is a significant factor in the selection of treatment [18].

## 2. Long Noncoding RNA

Genetic studies have found that only 2% of human genomic DNA can be subjected to transcription into mRNA, while the remaining 98% can be transcribed into noncoding RNA [19,20]. With the advancement of scientific research techniques, a growing number of lncRNA have been discovered. Long noncoding RNA (lncRNA) is an RNA sequence, with a length not exceeding 200 nucleotides, which does not code any polypeptide or protein. The actions of lncRNAs are based on their sponging and sequestering miRNAs [21]. This way, they hamper the interaction of miRNA with target mRNA [22,23].

Recently, lncRNAs’ function as vital regulators of numerous cellular processes has been discovered. They are believed to be involved in various physiological or pathological processes [24,25]. LncRNA can modulate genes at three levels: epigenetic, transcriptional, and post-transcriptional [20]. These RNAs are involved in numerous diseases, including the development, progression, and metastasis of cancers [21,26,27]. The role of lncRNAs in the modulation of cancer occurrence and development could involve their actions in the regulation of gene expression [28]. They have been found to stimulate proliferative signalling, enable the evading of immune destruction and surveillance, promote replicative immortality, induce angiogenesis, and act as a trigger for invasion and metastasis [14,16,29]. LncRNAs are involved in many cellular functions, the majority of which require interaction with proteins [13]. The results of many studies have shown that lncRNAs regulate protein stability via RNA-protein interactions [30,31]. Growing evidence indicates that such lncRNAs can be used as prognostic biomarkers [32].

Tumour cells adjust their metabolic pathways to survive as well as to grow and progress in rough tumour microenvironments (TMEs). Therefore, they switch glucose metabolism from phosphorylation to aerobic glycolysis (Warburg effect) [33]. The metabolites obtained in the course of glycolysis are used by cancer cells to fuel proliferation and metastasis. In turn, secreted lactate, which forms an acidic microenvironment, stimulates tumour cell invasion and escape from chemotherapy [34,35]. Abnormally enhanced glycolysis has been suggested to correlate with a poor prognosis of ccRCC. Thus, it appears that glycolysis may be used as a potential target for the diagnosis and therapy of ccRCC. Li et al. [26] identified three glycolysis-related lncRNAs (AC156455.1, AC009084.1, and LINC00342) which enabled the prediction of ccRCC patients’ prognosis. Based on such lncRNA signatures, authors were able to divide ccRCC patients into high- and low-risk groups with high sensitivity and specificity. This signature correlated with poor overall survival of patients with ccRCC in various ages, tumour grades, and clinical stage groups. It appears that the aforementioned glycolysis-related lncRNA signature could support the prediction of a clinical prognosis. LncRNA has been suggested to modulate the expression of glycolytic enzymes which are responsible for the stimulation of glycolytic levels and progression in tumour cells. Being able to interact with transcription factor receptors (aryl hydrocarbon receptor), lncRNAs enhance the transcription of important glycolytic enzymes (i.e., hexokinase 2 or pyruvate kinase M2) thus facilitating tumour cell progression [36,37]. Moreover, they can inhibit the degradation of some enzymes via the impact on ubiquitination [38]. LncRNA-induced modulation of glycolytic levels and other glycolysis-related pathways can be also based on the interactions with specific micro-RNAs (miRNA). Many lncRNA acts as miRNA sponges or competing RNA. The inhibition of targeted miRNAs translates into the alterations of glycolysis. Zhao et al. [39] demonstrated that lncRNA can even mediate glycolysis-associated positive feedback circuits resulting in glycolytic reprogramming towards persistent glycolysis and enhanced invasion and liver metastasis in colorectal cancer.

The occurrence of cancers is associated with mutations in DNA repair genes which results in genomic instability (GI) [40]. Genomic instability is the source of the heterogeneity within and between tumours [41]. Heterogeneity affecting key tumour pathways and inducing phenotypic variation is associated with the progression and prognosis of tumours [42,43]. The results of studies have indicated that long noncoding RNAs are involved in the maintenance of genomic stability [44]. Single nucleotide variants (SNVs) were found to be prevalent in tumours and occur within noncoding RNA, especially lncRNA, resulting in their aberrant function or expression [45,46]. The term genomic instability applies to DNA sequence variations, the instability of chromosomes, and chromatin’s higher-order structure [47]. LncRNAs have been demonstrated to regulate the mitotic checkpoint and centromere proteins which could result in the formation of aneuploidy [48,49]. Chromosomal instability and tumorigenesis were suggested to be associated with the upregulation of telomeric repeat-containing lncRNA and subsequent stabilization of shortened telomeres [44,50]. Moreover, the disruption of topologically associated domains (TADs) via genomic rearrangements can lead to aberrant gene misexpression and the development of disease [51]. Properly functioning lncRNA can prevent TADs damage since they help to preserve TADs stability [52,53]. Also, DNA disruption, particularly alterations resulting in defective DNA double-strand breaks (DSB) repair, is associated with tumorigenesis [54]. The results of studies have indicated that some damage-induced lncRNAs can be considered a key regulator in DSB misrepair, thus leading to genomic instability and the development of cancers. The role of GI-derived lncRNAs in RCC has not been widely studied, however, one of the studies suggested that the following lncRNAs, LINC00460, AL139351.1, AC156455.1, AL035446.1, LINC02471, AC022509.2, and LINC01606, may be involved in the cell cycle, the replication of DNA, mismatch repair, and consequently in the development and progression of ccRCC [44]. However, so far, some of these RNAs have not been associated with any tumours.

Another study has suggested that the lncRNA signature is closely correlated with distant metastasis in ccRCC and prognosis [55]. This signature involved LINC01234, LINC02577, and LINC02609. The first lncRNA was reported to be markedly upregulated also in some other cancers. However, no reports are available concerning the role of LINC02577 and LINC02609 in human cancers. In the study performed by Su et al. [55], the aforementioned lncRNA promoted distant metastasis of ccRCC via the mechanism involving the regulation of autophagy. The authors suggested that a set of distant metastasis-related lncRNAs could serve not only as a reliable prognosis biomarker but also as a promising therapeutic target for ccRCC. However, first, their utility has to be verified in larger studies.

**Figure 1:**
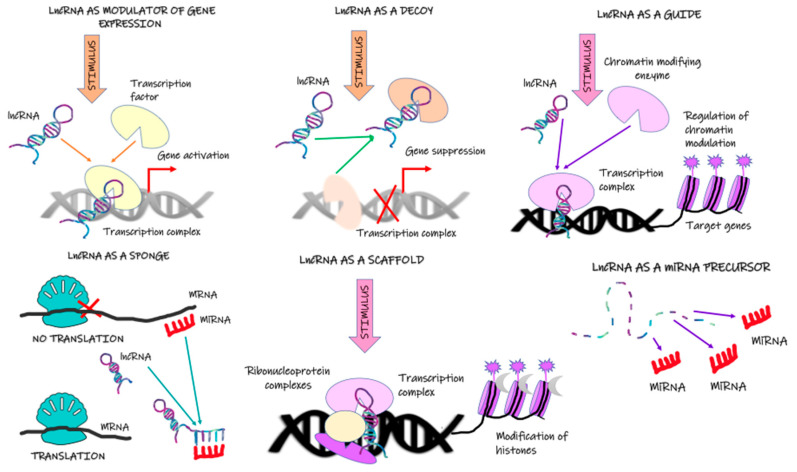
Mechanisms of lncRNA action.

Hierarchical clustering analysis demonstrated that patients with progressive disease and nonprogressive disease have differing transcriptome levels, which allows identifying new prospective biomarkers [17]. However, the mechanisms responsible for the abnormal expression of lncRNAs, including upstream regulatory mechanisms in cancer cells, have not been well established. Available scientific data suggest that lncRNA expression is regulated by transcription factors, histone status, DNA methylation patterns, and post-transcriptional regulation [56]. Zang et al. [57] demonstrated that lncRNA CCAT1 activation depends on H3K27 acetylation. In turn, enhanced expression of lncRNA H19 is associated with reduced methylation of the CpG islands in the promoter region [58]. In the case of other lncRNA (lnc01503), its increased expression stemmed from the greater capacity of the transcription factor to bind to the promoter region [59]. Hammerle et al. [60] showed that the interaction between IGF2BP1 and the lncRNA HULC not only diminished the stability of the latter, it also diminished its expression. The lack of sound data in this field requires further research. Mechanisms of lncRNA action are presented in Figure 1.

Below, we mention some of the lncRNA with a potential role in the metastasis and prognosis of ccRCC.

### 2.1. MALAT1

Recently, extracellular vesicles (EVs) have been attracting attention due to their potential role in tumour development and progression [61]. They are composed of exosomes and microvesicles, which are involved in intercellular communication [62]. According to many studies, the ability of tumour cells to secrete EV correlates with their capacity to metastasize from a primary tumor [63,64,65,66]. Secreted EV spread from the primary tumor via body fluids, and when they reach distant organs, they promote the formation of a pre-metastatic niche. The EVs contain functional cargo which enables them to modify the phenotype of receiving cells as well as extracellular matrix ECM organization [67,68,69]. Tumour EVs were found to affect various mechanisms of tumour progression, including proliferation, endothelial permeability, invasion, immune response, and drug resistance [69,70,71]. Aggressive tumour cells deprived of the genes responsible for the secretion of EV (such as RalA, RalB, nSMase2, Rab27a) were less likely to metastasize [72,73,74]. The exposure of A498 and ACHN cells to RCC cell 786-O-derived EVs was found to improve cell viability, migration, and invasiveness, as well as stimulate epithelial-mesenchymal transition (EMT) [61]. Extracellular vesicles’ ability to facilitate tumorigenesis is related to their involvement in the transfer of various RNAs, including lncRNAs [75]. Many studies have confirmed the role of tumour-derived extracellular vesicles in the progression and metastasis-related stimulation of premetastatic niche preparation [61,76]. One lncRNA, metastasis-associated lung adenocarcinoma transcript 1 (MALAT1), also known as nuclear-enriched abundant transcript two, has been suggested to be not only the marker of serious pathological changes in cancerous organs, but also to promote tumorigenesis [77]. MALAT1 transcripts have a length of approximately 8000 nucleotides and this lncRNA is highly conserved among mammals [78,79]. Hirata et al. [80] demonstrated that transcriptional activation of MALAT1 by Fos promoted the oncogenesis, while its knockdown translated into suppressed oncogenic properties and depleted Ezh2, and subsequent EMT inhibition resulting from the recovery of E-Cadherin and beta-catenin down regulation. The aforementioned Ezh2 boosts methylation of the H3K27 gene involved in cancer progression and metastasis which leads to its silencing [81]. An in vitro study confirmed reduced cell invasion and enhanced apoptosis in RCC cell lines with MALAT1 knocked down [80]. LncRNA MALAT1 was also found to stimulate the expression of Livin protein by improving its stability [82]. Interaction between MALAT-1 and Livin was demonstrated to be crucial for RCC cell viability and cell apoptosis. Another study revealed that this oncogene was associated with poor survival in various RCC specimens [83]. Numerous binding sites for EST1 have been identified within the TFCP2L1 gene promoter. Aberrant overexpression of MALAT1 was demonstrated to be an oncogenic mediator in RCC [80]. Increased ETS1, resulting from lncRNA-mediated regulation, favours malignant behaviours and tumour growth in ccRCC [84]. Thus, it appears that MALAT1 negatively modulates TFCP2L1 through the specific binding to ETS1. Following the binding of MALAT1 with ETS1 protein, a complex is formed, and it participates in transcriptional regulation. Predominant MALAT1 expression was found in extracellular vesicles secreted by various cancer cells. Following the release, such EVs become transported to recipient cells to promote the progression of the tumour [85,86]. Several studies have confirmed the strict relationship between elevated levels of MALAT1 and improved viability, migration, and invasion of RCC cells [61,87]. In turn, the knockdown of MALAT1 hampered the malignant behaviours of RCC cells [88]. In nude mice with RCC, the lack of MALAT1 was associated with significant suppression of xenograft growth [82]. Some studies have suggested the involvement of MALAT1 in epithelial-mesenchymal transition (EMT) [89]. Other studies have suggested that the predominant mechanism associated with MALAT1 function involves the regulation of RNA processing [90]. According to Tripathi et al. [90], MALAT1 controls RNA splicing via interaction with many splicing factors, including serine/arginine-rich splicing factor one and serine/arginine-rich splicing factor three. MALAT1 can also bind numerous subunits of the RNA spliceosome (e.g., ATP-dependent RNA helicase A, serine/arginine-rich splicing factor 7, and splicing factor U2AF2 [91]). Moreover, it was found that MALAT1 could interact with various pre-mRNAs, for example, precofilin-1 (pre-CFL1) via specific proteins [92]. Furthermore, one of the studies indicated the relationship between CFL1 and cell invasion and cancer metastasis [93,94]. Zhang et al. [89] demonstrated that the absence of MALAT1 was associated with reduced CFL1 mRNA expression and lower protein level, however, the level of pre-mRNA remained unchanged. This finding could be explained by the fact that it is probably MALAT1 that affects the alternative splicing of pre-CFL1, which leads to the enhanced formation of unstable CFL1 transcripts that immediately undergo degradation. Moreover, they revealed that MALAT1 can promote cell migration and invasion via a mechanism related to the alteration of CFL1 expression in RCC cells and subsequent change of F-actin level [89].

### 2.2. RCAT1

RCAT1 (renal cancer-associated transcript one or ENSG00000270661) has recently been recognised as an important tumour promoter in RCC [95]. The expression of this lncRNA was found to be markedly upregulated in RCC tissues. It also correlated with the poor prognosis of RCC patients. Due to its cytoplasmic distribution, ENSG00000270661 can serve as a ceRNA. The analysis of mechanisms through which lncRNA RCAT1 could promote ccRCC cell proliferation, migration, and invasion revealed that it can sponge miR-214-5p [95]. Moreover, lncRNA RCAT1 knockdown was associated with a higher expression of miR-214-5p, while its overexpression led to downregulation of miR-214-5p in renal cancer cells, thus suggesting the presence of a negative regulatory effect. Many studies have demonstrated that miR-214-5p plays a tumour-suppressive role in various cancers [96,97]. The overexpression of this miRNA was found to prevent the proliferation and metastasis of RCC cells [95]. Furthermore, lncRNA RCAT1-mediated malignant behaviour was partly limited by miR-214-5p. Since no information on the protein-binding potential of lncRNA RCAT1 and related mechanisms is available, this issue requires further studies. Based on the role of various lncRNA in cancer cells, it has been hypothesized that lncRNA RCAT1 might play a putative role in transcriptional processing with a protein-binding potential to stimulate the progression of tumours [95]. Since lncRNA RCAT1 serves as ceRNA, it has been hypothesized that the PI3K–Akt signalling pathway may be involved in its biological effects. In general, the PI3K–Akt signalling pathway is considered to be important for cell proliferation, apoptosis, and motility [98]. The results of studies have revealed constitutive activation of this signalling pathway in ccRCC, which was associated with cancer progression [99,100].

Owing to advancements in molecular biology and genetics techniques, targeting lncRNA seems to be a promising therapy. The use of antisense oligonucleotides and RNA interference (RNAi) therapy may in the future enable the control of tumour growth, development, and metastasis. The experiments involving the delivery of nanoparticle-mediated RNAi targeting other oncogenic lncRNA (DANCT) have brought encouraging results as they indicated effective cellular uptake, prolonged target silencing, as well as the lack of evident toxic side effects [101].

### 2.3. DUXAP9

LncRNA DUXAP9 (ENSG00000225210) is another molecule possibly related to tumorigenesis. According to studies, DUXAP9 is m6A modified and binds to IGF2BP2, which raises its stability [102]. The interaction of lncRNA with IGF2BP2 modulates a variety of biological mechanisms [100,103]. IGF2BPS, which belongs to the family of m6A card readers, can bind to a specific m6A sequence, thus targeting numerous transcripts. The presence of m6A modifications in lncRNAs has been suggested to be implicated in the development of various cancers [13]. M6A methylation is the predominant post-transcriptional RNA modification occurring in the majority of eukaryotic cells. This process is reversible, and it is catalysed by enzymes, known as “writers,” “erasers,” and “readers.” Such modifications have been linked with cell proliferation, differentiation, and tumorigenesis [104,105]. Tan et al. [102] revealed a vital impact of m6A modification on the effects of DUXAP9. Indeed, the silencing of either METTL3 or IGF2BP2 was associated with lower RNA stability resulting from the inhibition of m6A modification and a consequent reduction in the level of DUXAP9. This finding implies that DUXAP9 and IGF2BP2 form an RNA–protein complex in RCC. It also indicates that the biological effects of DUXAP9 depend on m6A modification [102]. Moreover, DUXAP9 was found to activate the PI3K/AKT pathway as well as the expression of Snail in localized ccRCC. The results of one of the studies indicate that DUXAP9 knockdown diminished the activation of Akt signalling in renal cancer cells. It was suggested that DUXAP9-mediated stimulation of proliferation involved the regulation of Akt/mTOR [102]. The inhibition of PI3K was demonstrated to abrogate the tumorigenic effects of DUXAP9 in RCC. Thus, it appears that DUXAP9 can induce Akt/mTOR signalling via the activation of PI3K. The regulation of Snail, a transcriptional repressor of epithelial genes by DUXAP9 was shown to promote EMT in renal cancer cells [106]. This process involved the activation of the Akt-GSK3β-Snail signalling pathway [102].

Another study found that lncRNA DUXAP9 could directly interact with E3 ubiquitin ligase Cbl-b (Casitas B-lineage lymphoma proto-oncogene-b) which resulted in the diminished degradation of the epidermal growth factor receptor (EGFR) [107]. Enhanced EGFR signalling was demonstrated to promote proliferation and metastasis in cancers. The oncogenic potential of DUXAP9 has been explored in various cancers, including thyroid cancer, nonsmall cell lung cancer, bladder cancer, and renal cell carcinoma [107,108,109]. Tan et al. [102] demonstrated significant upregulation of lncRNA DUXAP9 in localized ccRCC. Moreover, they found a correlation between the expression of this RNA, the overall survival, and the progression-free survival (PFS) in ccRCC patients.

### 2.4. LncRNA TCL6 (lncTCL6)

For the first time, lncRNA TCL6 has been reported in T-cell leukaemia [110]. It was located in the vicinity of the TCL1B protein-coding gene on chromosome 14q32.1. More recent studies indicate its independent predictive value of ccRCC aggressiveness [111,112]. Kulkarni et al. [110] demonstrated an inverse correlation between the levels of lncTCL6, more advanced tumour grade, and decreased overall survival. Moreover, the analysis of its expression enables the differentiation between cancerous/noncancerous tissues in RCC. Thus, it can serve as a diagnostic biomarker in ccRCC.

According to suggestions, TCL6 may regulate the EGFR/AKT pathway in placental tissue [113]. The results of Kulkarni et al. [110] research point to lncRNA and miRNA-mediated regulation of the Src-Akt pathway that triggers metastasis in renal cancer. LncTCL6 was found to be the miR-155 target. According to the authors, the lncTCL6-miR-155-Src/Akt/EMT network poses a new regulatory mechanism related to ccRCC progression and metastasis. They demonstrated markedly downregulated lncTCL6 and considerably upregulated miR-155-5p in renal cancer tissues. Such a profile was positively correlated with poor overall survival of RCC patients [110]. LncTCL6 was found to exert tumour-suppressive effects in renal cancer since its overexpression limited the Src-Akt-mediated metastatic process as a result of the recruitment of STAU1 protein to Src mRNAs and subsequent decay of the latter. In many cancers, the activation of Akt and Src correlated with the presence of malignant phenotypes and worse survival in renal carcinoma [114,115]. In other studies, lncTCL6, acting as a tumour-suppressor, improved overall survival [111,116,117]. In vitro overexpression of lncTCL6 was found to markedly reduce the proliferation of ccRCC cells, the formation of colonies, and migration of tumour cells, as well as invasion, G2/M arrest, and apoptosis [110]. Opposite effects were observed in the case of miR-155 overexpression as well as lncTCL6 suppression. In this study, greater aggressiveness of renal cell cancer involved the activation of Src-Akt-induced EMT triggered via miR-155 targeting lncTCL6 [110]. It appears that the interaction between lncTCL6 and miR-155 may be a promising target for a therapeutic approach in ccRCC.

### 2.5. LINC00342

Many studies have demonstrated that INC00342 acts as an oncogene. They indicated the association between its level and cancer progression in different cancers. LINC00342 was found to sponge miR-19a-3p, which was associated with the modulation of NPEPL1 expression and contributed to the growth and metastasis of colorectal cancer cells [118]. Li et al. [26] observed the correlation between the overexpression of LINC00342 and the poor prognosis of patients with ccRCC. In vitro study revealed its higher expression in ccRCC cell lines compared to human renal tubular epithelial cells. The silencing of LINC00342 with siRNA was found to reduce both the glycolytic level and migration capabilities of 786-O cells. These findings confirmed the vital role of LINC00342 in the reprogramming of glucose metabolism and metastasis. Some studies have suggested that abnormal tumour glycolytic levels may modify the crosstalk between tumour cells and the tumour microenvironment. This can result in immune resistance or escaping immune surveillance [119,120]. Indeed, lncRNA could stimulate tumour aerobic glycolysis and increase its resistance to immune immunotherapy [121]. In the study conducted by Li et al. [26], glycolysis-related lncRNA signature was associated with the infiltration of memory B cells, regulatory T cells, follicular helper T cell, and M0 macrophages. In turn, the distribution of monocytes, naive B cells, M0 macrophages, resting and activated dendritic cells, and resting mast cells was repressed in high-risk patients. In this group, T cell costimulation became activated, while type II IFN response was inactivated [26]. Such a signature may be associated with the arrest of immune response and contribute to ccRCC development. Moreover, they demonstrated that the silencing of LINC00342 was associated with diminished glycolytic levels and suppressed Wnt/β-catenin signalling pathway. This signalling pathway was found to be involved in the Warburg effect and the malignant progression of cancer cells [122,123]. Moreover, it can promote the rise in glucose uptake and limit mitochondrial respiration favouring the proliferation of cancer cells [124].

### 2.6. AGAP2-AS1 (AGAP2 Antisense 1)

This RNA, known also as PUNISHER ENSG00000255737, is located at 12q14.1 [125]. Nakken et al. [17] have studied the expression profiles in ccRCC patients who, despite being classified as having a low risk of progression, developed metastasis during a follow-up period [17]. They found that the expression level of noncoding RNA AGAP2-AS1 in tumour tissues appropriately predicted 100% of the nonprogressor group and nearly 90% of the progressor group, already from the time of surgery. Higher expression of AGAP2-AS1 was reported in patients whose disease progressed compared to nonprogressors. The same results were obtained by a qPCR analysis of serum samples. However, this time, the differences were not significant, probably due to too small a cohort. Confirmation of observed differences would enable the use of liquid biopsies instead of solid tissue samples for diagnostic purposes [126]. Interestingly, Nakken et al. [17] failed to demonstrate significant differences in AGAP2-AS1 expression between the original tumour and metastases, of which biopsy was made on average 4.5 years later. This indicates that the expression of AGAP2-AS1 is stable over time, which supports its reliability as a biomarker. The upregulation of AGAP2-AS1 was also reported in other cancers which correlated with lower survival rates [127,128]. The role of AGAP2-AS1 in tumours was confirmed by studies revealing that its silencing limited the proliferation and invasion potential as well as enhanced tumour cell apoptosis [129,130,131,132]. Furthermore, the AGAP2-AS1 gene knockdown was associated with restored sensitivity to treatment with trastuzumab in breast cancer cell lines previously overexpressing AGAP2-AS1 and resistant to this therapy [133]. Another study has also shown higher AGAP2-AS1 expression in metastatic cancer tissues compared to localized prostate cancer [134].

The results of studies revealed that AGAP2-AS1 was coexpressed with HDGF and ANGPTL4, both of which were related to tumour angiogenesis [125,130,135]. High levels of lncRNA AGAP2-AS1 considerably correlated with a worse survival status in ccRCC patients. The association between high AGAP2-AS1 expression and overall survival was observed in patients with histological grade G1/G2, grade G3/G4, and clinical stage III/IV, but not in individuals with histological clinical stage I/II. This finding shows the specific prognostic role of AGAP2-AS1 expression levels and provides a potential target for precision therapy for ccRCC [125]. Increased expression was associated with gender, age, more advanced clinical stage, and TNM stage in patients with ccRCC [125]. Mechanisms behind poor overall survival in patients with higher AGAP2-AS1 expression may involve angiogenesis, hypoxia, epithelial-mesenchymal transition, the notch signalling pathway, or stromal simulation [125,136,137,138]. Epithelial-mesenchymal transition, angiogenesis, and hypoxia are well-established characteristic features of cancers. Angiogenesis was found to play a vital role in the progression of ccRCC via a process involving VEGF, PDGF, FGF-2, chemokines, angiopoietins, apelin (APLN), and ephrins [139,140]. Gao et al. [125] demonstrated that increased expression of lncRNA AGAP2-AS1 was important for angiogenesis and suggested that the VEGF and Akt pathways might be involved in its actions. Further analyses revealed a significant correlation between elevated AGAP2-AS1 expression and G1/G2, stage I/II, and M0 cases.

### 2.7. DLEU2

DLEU2 is another long noncoding RNA whose aberrant expression was observed in various cancers [141]. Studies of different cancers have indicated the role of lncRNA in the evolution and progression of malignancies [142]. Since it was found for the first time in immune cell-associated disease (diffuse large B-cell lymphoma), it has been hypothesized that DLEU2 expression may correlate with immune cell infiltration [143]. According to studies, this RNA is involved in the progression of tumours through the regulation of EMT, the impact on the Akt signalling pathway, and the stimulation of tumour cell proliferation via modulating the Notch signalling pathway [144,145,146]. Fu et al. [141] demonstrated a higher expression of DLEU2 in ccRCC patients compared to matched normal samples. The aberrant expression of this long noncoding RNA was found to be associated with copy number variations and DNA methylation. The expression level correlated with the TNM stage of the tumor as well as with the immune markers of B cells, CD8+ T cells, dendritic cells, neutrophils, M2 macrophages, monocytes, T cell exhaustion, Th1 cells, Tfh cells, and Tregs. A relationship was observed between DLEU2, TGFB1, and CTLA4, which are involved in vital immunoregulatory activities. TGFB1 stimulates the expression of CTLA4, thus aggravating CTLA4-related inhibition of t-cell proliferation and cytokines release and promoting t-cell apoptosis, which enables tumour immune escape [147]. T-cells are known to play a key role in immune regulation in the tumour environment. According to studies, Tregs can reduce the immune system’s activity towards tumours and raise the body’s immune tolerance to them [148]. In turn, DC was found to stimulate tumour growth and metastasis as a result of rising Tregs and triggering CD8+ T cells [149]. Finally, M2 tumour-associated macrophages can facilitate the EMT process in tumours and enhance tumour cell activity [150]. DLEU2 expression was also found to be markedly associated with molecular markers of different tumour-infiltrating immune cell subtypes, such as CD8+ T cell markers, thus it appears that DLEU2 may modulate immune infiltration [141]. Moreover, it was found to modulate the proliferation and differentiation of immune cells [151]. It has been suggested that DLEU2 favours tumour development since it modulates the infiltration of immune cells in the TME [141]. In patients with ccRCC and high expression of DLEU2, the immune infiltration may, at least to some extent, modulate the prognosis. The analysis of the ROC curve indicates that DLEU2 can be used to predict the presence of ccRCC as well as disease progression. Moreover, Fu et al. [141] suggested that a high expression of this RNA was associated with a worse prognosis. The results of other studies also demonstrated that DLEU2 expression could stimulate tumour growth, spreading, and invasion in various types of cancers [144,152].

### 2.8. NNT-AS1

The expression of NNT-AS1 has been demonstrated in various cancers. It can serve as a sponge of miR-22, thus contributing to tumour progression [153]. Another study indicated that it increased the resistance of cervical cancer to cisplatin treatment [154]. However, the role of NNT-AS1 in ccRCC is not so well studied. Zhou et al. [20] found marked upregulation of NNT-AS1 in specimens collected from ccRCC compared to normal, healthy tissues. These authors suggested that the RNA may act as an oncogene related to the progression of ccRCC. While searching for the mechanism laying behind the neoplastic effects of NNT-AS1, they found that it can serve as a sponge to miR-137, which in turn, targets oncogene YBX-1. The silencing of NNT-AS1 was demonstrated to be associated with considerably limited ccRCC proliferation and metastasis. In turn, overexpression of NNT-AS1 stimulated both proliferation and invasion. The localisation of NNT-AS1 in cells (cytoplasm) may imply that it is involved in the regulation of gene expression at the post-transcriptional level. This hypothesis is supported by the fact that it can specifically bind with AGO2, a member of the Argonaute family. AGO2 is involved in small RNA-induced post-transcriptional gene silencing [155,156]. In order to exert its functions, NNT-AS1 binds to miRNA and modulates its expression. MiR-137 was found to be a downstream target of NNT-AS1. The silencing of this RNA enhanced miR-137 expression, while its overexpression brought the opposite effects. An in vivo study demonstrated an inverse correlation between miR-137 and NNT-AS1. Inhibition of miR-137 was associated with enhanced expression of YBX-1 at the mRNA and protein levels. Therefore, it seems that NNT-AS1 modulates the miR-137/YBX-1 axis, thus contributing to ccRCC progression [20].

### 2.9. LINC00460

In ccRCC cells and tissues, the upregulation of LINC00460 has been reported [21]. The role of LINC00460 has been studied in numerous tumours. For example, in nonsmall cell lung cancer (NSCLC), this lncRNA triggered epithelia-mesenchymal transition, cell migration, and tumour invasion, while in colorectal cancer it stimulated the resistance to cancer treatment [157,158,159]. LINC00460 has also been found to be associated with RCC. The determination of its expression level enabled the differentiation between normal renal cells and ccRCC cells [21]. Elevated LINC00460 expression significantly correlated with TNM stages and lymph node metastasis and was associated with a poor prognosis in RCC patients. The results of studies demonstrated that high LINC00460 expression stimulated the proliferation, migration, and invasion of ccRCC cells [21,160]. Therefore, it appears that this RNA can serve as a therapeutic target for patients with ccRCC as well as a prognostic biomarker. This thesis is supported by the results of studies demonstrating that the inhibition/down-regulation of LINC00460 was associated with hampered tumour cell proliferation and invasion in various cancers, such as colorectal cancer, gastric cancer, etc. [161,162]. An in vitro study of ccRCC cells confirmed the suppression of the proliferation, migration, and invasion when they were transfected with small interfering RNA (siRNA) for LINC00460 that knocks down LINC00460 [21]. The biological functions of this long noncoding RNA were suggested to be related to its ability to act as a ceRNA decoy for selected tumour-suppressive miRNAs. Further studies identified that miR-149-5p is the downstream target for LINC00460. MiR-149-5p directly regulates forkhead box protein M1 (FOXM1), which is a proliferation-related transcription factor, involved also in tumorigenesis [163]. It has been demonstrated to promote proliferation and cell cycle progression, and it is also necessary for the proper execution of mitosis. The overexpression of FOXM1 in cancers has been linked to oncogenic transformation, tumour initiation, growth, progression, migration, invasion, and metastasis via impact on epithelial-mesenchymal transition, angiogenesis, the recruitment of tumour-associated macrophages, the prevention of premature cellular senescence, and chemotherapeutic drug resistance [163]. The downregulation of this miRNA observed in ccRCC tissues and associated with the sponge effect from LINC00460 translated into the survival of patients with ccRCC [164,165]. To sum up, LINC00460 might favour ccRCC development and progression by sponging miR-149-5p thus affecting FOXM1 [21].

### 2.10. Lnc-LSG1

The expression of Lnc-LSG1 is prevalent in the cytoplasm, and to a lower extent, in the nucleus [13]. This RNA locus acts locally (in *cis*) to trigger the transcription of nearby genes via the stimulation of chromatin looping [166]. In order to exert its functions, lnc-LSG1 frequently requires interaction with proteins. Proteins potentially associated with this RNA have been summarized in the online Database catRAPID [167]. ESRP2 which modulates alternative splicing in epithelial cells is one of them. Shen et al. [13] demonstrated that Lnc-LSG1 may stimulate ccRCC metastasis via an epithelial-specific splicing regulator (ESRP2). Mizutani et al. [168] found that ESRP2 and Arkadia (also known as RNF111) inhibited ccRCC tumour growth in a coordinated manner. In their study, the overexpression of Lnc-LSG1 considerably reduced the level of ESRP2 protein, while the knockdown of Lnc-LSG1 was associated with higher ESRP2 protein levels. According to the authors, Lnc-LSG1 can bind directly to the ESRP2 protein resulting in ubiquitin-proteasome pathway-mediated inhibition of its expression, which translates into the stimulation of ccRCC metastasis. Binding with Lnc-LSG1 shortens the ESRP2 half life and leads to its degradation via the ubiquitination pathway [13]. Moreover, Lnc-LSG1 was found to be the downstream target of METTL14. METTL14, which exerts an antimetastatic effect on ccRCC cells, was shown to recruit YTHDC1 to a GGACU motif present on Lnc-LSG1 which in consequence hampered the binding between Lnc-LSG1 and ESRP2 protein and enhanced ESRP2 protein stability [13].

Table 1 presents the summary of the possible role of lncRNA in the progression of ccRCC cancer and metastasis.

## 3. Conclusions

All the aforementioned studies indicated the plausible role of lncRNA in the progression of ccRCC and metastasis. Some of them appear to be useful as biomarkers, factors enabling the prediction of disease course (progressive vs. nonprogressive), or differentiation between ccRCC and healthy tissue. However, before the expression of selected lncRNA could be used in clinical practice, their reliability has to be confirmed in large trials. Currently, there are just a few available research articles presenting the role of lncRNA in ccRCC, and therefore, it is hard to draw solid conclusions.

## Figures and Tables

**Table 1:** Possible role of lncRNA in the progression of ccRCC cancer and metastasis.

Name	Plausible Mechanisms Involved	Effects
LINC00342	Sponges miR-19a-3p [118]	➢ Correlation between its overexpression and poor prognosis of patients with ccRCC [26] ➢ Vital role in the reprogramming of glucose metabolism and metastasis ➢ Relation to the arrest of immune response ➢ Promotion of ccRCC development ➢ Wnt/β-catenin signalling pathway [26]
Lnc-LSG1	Reduction of ESRP2 protein level [13]	➢ Stimulation of ccRCC metastasis [13]
AGAP2-AS1	VEGF and Akt pathway Involvement of AGAP2-AS1 in angiogenesis, hypoxia, epithelial-mesenchymal transition, the notch signalling pathway, or stromal simulation [125,136,137,138]	➢ Higher expression in patients with progressive disease [17] ➢ Its expression level enabled the differentiation between the nonprogressor and progressor groups [17] ➢ AGAP2-AS1 expression is stable over time ➢ The upregulation correlated with lower survival rates [68,69] ➢ Its silencing limited the proliferation and invasion potential as well as enhanced tumour cell apoptosis [70,71,72,73].
DLEU2	Regulation of EMT, impact on Akt signalling pathway, stimulation of tumour cell proliferation via modulating Notch signalling pathway [144,145,146]	➢ DLEU2 expression may correlate with immune cell infiltration [143] ➢ Higher expression of DLEU2 in ccRCC patients compared to matched normal samples [141] ➢ Correlation between expression level and TNM stage of tumour and immune markers [141] ➢ DLEU2 modulates the proliferation and differentiation of immune cells [92] ➢ DLEU2 expression could stimulate tumour growth, spreading, and invasion [85,93]
NNT-AS1	Sponge of miR-22, miR-137 possible involvement in the regulation of gene expression at the post-transcriptional level [20] modulation of miR-137/YBX-1 axis	➢ Contribute to tumour progression [153] ➢ Upregulation of NNT-AS1 in ccRCC compared to healthy tissues [20] ➢ Silencing of NNT-AS1 results in considerably limited ccRCC proliferation and metastasis [20]
LINC00460	MiR-149-5p—the downstream target for LINC00460 modulation of FOXM1	➢ Upregulation of LINC00460 in ccRCC cells and tissues [21] ➢ Expression level enables the differentiation between normal renal cells and ccRCC cells [21] ➢ Elevated expression correlated with TNM stages and lymph node metastasis [21] ➢ suppression of the proliferation, migration, and invasion in cells transfected with siRNA that knockdown LINC00460 [21]
MALAT1	Transcription factor (ETS1) Involvement in the regulation of RNA processing [90] Interaction with precofilin-1 (pre-CFL1) [92].	➢ Promotion of tumourigenesis [77] ➢ Stimulation of RCC invasion and metastasis ➢ Expression is associated with poor survival [83]. ➢ Elevated levels of MALAT1 improved viability, migration, and invasion of RCC cells [61,87] ➢ Knockdown of MALAT1 hampers malignant behaviours [88]
RCAT1	Sponge of miR-214-5p	➢ Markedly upregulated expression in RCC tissues [95]. ➢ Positive correlation with poor prognosis of RCC patients [95] ➢ Stimulation of ccRCC cell proliferation, migration, and invasion [95]
DUXAP9	m6A modifications, binding to IGF2BPS, the activation of the Akt-GSK3β-Snail signalling pathway, the interaction with E3 ubiquitin ligase Cbl-b [102] Induction of Akt/mTOR signalling via the activation of PI3K [102]	➢ Significant upregulation in localized ccRCC [102] ➢ Promotion of EMT in renal cancer cells [141] ➢ DUXAP9-related enhanced EGFR signalling promotes proliferation and metastasis in cancers [107]. ➢ Correlation between its expression and the overall survival and progression-free survival in ccRCC patients [102].
TCL6	Regulation of EGFR/AKT pathway [113] lncTCL6-miR-155-Src/Akt/EMT network [110]	➢ Marked downregulation of lncTCL6 and considerable upregulation miR-155-5p in in renal cancer tissues [145] ➢ Inverse correlation between the levels of lncTCL6 and more advanced tumour grade and decreased overall survival [145]. ➢ Its expression enables the differentiation between cancerous/non-cancerous tissues in RCC [145] ➢ Its overexpression limits the Src-Akt-mediated metastatic process [110] ➢ Its overexpression significantly reduces the proliferation of ccRCC cells, the formation of colonies, migration of tumour cells as well as invasion, G2/M arrest, and apoptosis [110]

## Data Availability

Not applicable.

## References

[1] Ljungberg B., Albiges L., Abu-Ghanem Y., Bensalah K., Dabestani S., Fernández-Pello S., Giles R.H., Hofmann F., Hora M., Kuczyk M.A. (2019). European Association of Urology Guidelines on Renal Cell Carcinoma: The 2019 Update. Eur. Urol..

[2] Siegel R.L., Miller K.D., Jemal A. (2020). Cancer statistics, 2020. CA Cancer J. Clin..

[3] Harada K.i., Miyake H., Kusuda Y., Fujisawa M. (2012). Expression of epithelial–mesenchymal transition markers in renal cell carcinoma: Impact on prognostic outcomes in patients undergoing radical nephrectomy. BJU Int..

[4] Capitanio U., Bensalah K., Bex A., Boorjian S.A., Bray F., Coleman J., Gore J.L., Sun M., Wood C., Russo P. (2019). Epidemiology of renal cell carcinoma. Eur. Urol..

[5] Torre L.A., Bray F., Siegel R.L., Ferlay J., Lortet-Tieulent J., Jemal A. (2015). Global cancer statistics, 2012. CA Cancer J Clin..

[6] Brannon A.R., Reddy A., Seiler M., Arreola A., Moore D.T., Pruthi R.S., Wallen E.M., Nielsen M.E., Liu H., Nathanson K.L. (2010). Molecular Stratification of Clear Cell Renal Cell Carcinoma by Consensus Clustering Reveals Distinct Subtypes and Survival Patterns. Genes Cancer.

[7] Haake S.M., Brooks S.A., Welsh E., Fulp W.J., Chen D.T., Dhillon J., Haura E., Sexton W., Spiess P.E., Pow-Sang J. (2016). Patients with ClearCode34-identified molecular subtypes of clear cell renal cell carcinoma represent unique populations with distinct comorbidities. Urol. Oncol..

[8] Ghatalia P., Rathmell W.K. (2018). Systematic Review: ClearCode 34—A Validated Prognostic Signature in Clear Cell Renal Cell Carcinoma (ccRCC). Kidney Cancer.

[9] Wang B., Chen D., Hua H. (2021). TBC1D3 family is a prognostic biomarker and correlates with immune infiltration in kidney renal clear cell carcinoma. Mol. Oncolytics.

[10] American Cancer Society (2019). Cancer Facts & Figures 2019.

[11] Pulikkottil A.J., Bamezai S., Ammer T., Mohr F., Feder K., Vegi N.M., Mandal T., Kohlhofer U., Quintanilla-Martinez L., Sinha A. (2021). TET3 promotes AML growth and epigenetically regulates glucose metabolism and leukemic stem cell associated pathways. Leukemia.

[12] Leibovich B.C., Blute M.L., Cheville J.C., Lohse C.M., Frank I., Kwon E.D., Weaver A.L., Parker A.S., Zincke H. (2003). Prediction of progression after radical nephrectomy for patients with clear cell renal cell carcinoma: A stratification tool for prospective clinical trials. Cancer.

[13] Shen D., Ding L., Lu Z., Wang R., Yu C., Wang H., Zheng Q., Wang X., Xu W., Yu H. (2022). METTL14-mediated Lnc-LSG1 m6A modification inhibits clear cell renal cell carcinoma metastasis via regulating ESRP2 ubiquitination. Mol. Nucleic Acids.

[14] Lin G., Wang H., Wu Y., Wang K., Li G. (2021). Hub Long Noncoding RNAs with m6A Modification for Signatures and Prognostic Values in Kidney Renal Clear Cell Carcinoma. Front. Mol. Biosci..

[15] Thomas J.S., Kabbinavar F. (2015). Metastatic clear cell renal cell carcinoma: A review of current therapies and novel immunotherapies. Crit. Rev. Oncol. Hematol..

[16] Gutschner T., Diederichs S. (2012). The hallmarks of cancer: A long non-coding RNA point of view. RNA Biol..

[17] Nakken S., Eikrem Ø., Marti H.P., Beisland C., Bostad L., Scherer A., Flatberg A., Beisvag V., Skandalou E., Furriol J. (2021). AGAP2-AS1 as a prognostic biomarker in low-risk clear cell renal cell carcinoma patients with progressing disease. Cancer Cell Int..

[18] Gallardo E., Méndez-Vidal M.J., Pérez-Gracia J.L., Sepúlveda-Sánchez J.M., Campayo M., Chirivella-González I., García-Del-Muro X., González-Del-Alba A., Grande E., Suárez C. (2018). SEOM clinical guideline for treatment of kidney cancer (2017). Clin. Transl. Oncol..

[19] Qian X., Zhao J., Yeung P.Y., Zhang Q.C., Kwok C.K. (2019). Revealing lncRNA structures and interactions by sequencing-based approaches. Trends Biochem. Sci..

[20] Zhou Y., Zhang Z., Wo M., Xu W. (2021). The long non-coding RNA NNT-AS1 promotes clear cell renal cell carcinoma progression via regulation of the miR-137/Y-box binding protein 1 axis. Bioengineered.

[21] Zhang S., Zhang F., Niu Y., Yu S. (2021). Aberration of lncRNA LINC00460 is a Promising Prognosis Factor and Associated with Progression of Clear Cell Renal Cell Carcinoma. Cancer Manag. Res..

[22] Shima H., Kida K., Adachi S., Yamada A., Sugae S., Narui K., Miyagi Y., Nishi M., Ryo A., Murata S. (2018). Lnc RNA H19 is associated with poor prognosis in breast cancer patients and promotes cancer stemness. Breast Cancer Res. Treat..

[23] Cabili M.N., Trapnell C., Goff L., Koziol M., Tazon-Vega B., Regev A., Rinn J.L. (2011). Integrative annotation of human large intergenic noncoding RNAs reveals global properties and specific subclasses. Genes Dev..

[24] Peng W.-X., Koirala P., Mo Y.-Y. (2017). LncRNA-mediated regulation of cell signaling in cancer. Oncogene.

[25] Zhao Z., Sun W., Guo Z., Zhang J., Yu H., Liu B. (2020). Mechanisms of lncRNA/microRNA interactions in angiogenesis. Life Sci..

[26] Li T., Tong H., Zhu J., Qin Z., Yin S., Sun Y., Liu X., He W. (2021). Identification of a Three-Glycolysis-Related lncRNA Signature Correlated With Prognosis and Metastasis in Clear Cell Renal Cell Carcinoma. Front. Med. (Lausanne).

[27] Xia R., Geng G., Yu X., Xu Z., Guo J., Liu H., Li N., Li Z., Li Y., Dai X. (2021). LINC01140 promotes the progression and tumor immune escape in lung cancer by sponging multiple microRNAs. J. Immunother. Cancer.

[28] Barth D.A., Slaby O., Klec C., Juracek J., Drula R., Calin G.A., Pichler M. (2019). Current concepts of non-coding RNAs in the pathogenesis of non-clear cell renal cell carcinoma. Cancers.

[29] Bach D.-H., Lee S.K. (2018). Long noncoding RNAs in cancer cells. Cancer Lett..

[30] McHugh C.A., Chen C.K., Chow A., Surka C.F., Tran C., McDonel P., Pandya-Jones A., Blanco M., Burghard C., Moradian A. (2015). The Xist lncRNA interacts directly with SHARP to silence transcription through HDAC3. Nature.

[31] Gupta R.A., Shah N., Wang K.C., Kim J., Horlings H.M., Wong D.J., Tsai M.C., Hung T., Argani P., Rinn J.L. (2010). Long non-coding RNA HOTAIR reprograms chromatin state to promote cancer metastasis. Nature.

[32] Chen H., Pan Y., Jin X., Chen G. (2021). Identification of a four hypoxia-associated long non-coding RNA signature and establishment of a nomogram predicting prognosis of clear cell renal cell carcinoma. Front. Oncol..

[33] Liberti M.V., Locasale J.W. (2016). The Warburg effect: How does it benefit cancer cells?. Trends Biochem. Sci..

[34] Uehara T., Doi H., Ishikawa K., Inada M., Tatsuno S., Wada Y., Oguma Y., Kawakami H., Nakamatsu K., Hosono M. (2021). Serum lactate dehydrogenase is a predictive biomarker in patients with oropharyngeal cancer undergoing radiotherapy: Retrospective study on predictive factors. Head Neck.

[35] Li H., Qi Z., Niu Y., Yang Y., Li M., Pang Y., Liu M., Cheng X., Xu M., Wang Z. (2021). FBP1 regulates proliferation, metastasis, and chemoresistance by participating in C-MYC/STAT3 signaling axis in ovarian cancer. Oncogene.

[36] Zhu S., Guo Y., Zhang X., Liu H., Yin M., Chen X., Peng C. (2021). Pyruvate kinase M2 (PKM2) in cancer and cancer therapeutics. Cancer Lett..

[37] Yan T., Shen C., Jiang P., Yu C., Guo F., Tian X., Zhu X., Lu S., Han B., Zhong M. (2021). Risk SNP-induced lncRNA-SLCC1 drives colorectal cancer through activating glycolysis signaling. Signal Transduct. Target. Ther..

[38] Liu C., Zhang Y., She X., Fan L., Li P., Feng J., Fu H., Liu Q., Liu Q., Zhao C. (2018). A cytoplasmic long noncoding RNA LINC00470 as a new AKT activator to mediate glioblastoma cell autophagy. J. Hematol. Oncol..

[39] Zhao S., Guan B., Mi Y., Shi D., Wei P., Gu Y., Cai S., Xu Y., Li X., Yan D. (2021). LncRNA MIR17HG promotes colorectal cancer liver metastasis by mediating a glycolysis-associated positive feedback circuit. Oncogene.

[40] Negrini S., Gorgoulis V.G., Halazonetis T.D. (2010). Genomic instability—An evolving hallmark of cancer. Nat. Rev. Mol. Cell Biol..

[41] Burrell R.A., McGranahan N., Bartek J., Swanton C. (2013). The causes and consequences of genetic heterogeneity in cancer evolution. Nature.

[42] Malihi P.D., Graf R.P., Rodriguez A., Ramesh N., Lee J., Sutton R., Jiles R., Ruiz Velasco C., Sei E., Kolatkar A. (2020). Single-Cell Circulating Tumor Cell Analysis Reveals Genomic Instability as a Distinctive Feature of Aggressive Prostate Cancer. Clin. Cancer Res..

[43] Suzuki K., Ohnami S., Tanabe C., Sasaki H., Yasuda J., Katai H., Yoshimura K., Terada M., Perucho M., Yoshida T. (2003). The genomic damage estimated by arbitrarily primed PCR DNA fingerprinting is useful for the prognosis of gastric cancer. Gastroenterology.

[44] Yang H., Xiong X., Li H. (2021). Development and Interpretation of a Genomic Instability Derived lncRNAs Based Risk Signature as a Predictor of Prognosis for Clear Cell Renal Cell Carcinoma Patients. Front. Oncol..

[45] Cheetham S.W., Gruhl F., Mattick J.S., Dinger M.E. (2013). Long noncoding RNAs and the genetics of cancer. Br. J. Cancer.

[46] Lin C., Yang L. (2018). Long Noncoding RNA in Cancer: Wiring Signaling Circuitry. Trends Cell Biol..

[47] Guo F., Li L., Yang W., Hu J.F., Cui J. (2021). Long noncoding RNA: A resident staff of genomic instability regulation in tumorigenesis. Cancer Lett..

[48] Panda S., Setia M., Kaur N., Shepal V., Arora V., Singh D.K., Mondal A., Teli A., Tathode M., Gajula R. (2018). Noncoding RNA Ginir functions as an oncogene by associating with centrosomal proteins. PLoS Biol..

[49] Deng X., Li S., Kong F., Ruan H., Xu X., Zhang X., Wu Z., Zhang L., Xu Y., Yuan H. (2020). Long noncoding RNA PiHL regulates p53 protein stability through GRWD1/RPL11/MDM2 axis in colorectal cancer. Theranostics.

[50] Deng Z., Wang Z., Xiang C., Molczan A., Baubet V., Conejo-Garcia J., Xu X., Lieberman P.M., Dahmane N. (2012). Formation of telomeric repeat-containing RNA (TERRA) foci in highly proliferating mouse cerebellar neuronal progenitors and medulloblastoma. J. Cell Sci..

[51] Lupiáñez D.G., Spielmann M., Mundlos S. (2016). Breaking TADs: How Alterations of Chromatin Domains Result in Disease. Trends Genet..

[52] Yamamoto T., Saitoh N. (2019). Non-coding RNAs and chromatin domains. Curr. Opin. Cell Biol..

[53] Xiang J.F., Yin Q.F., Chen T., Zhang Y., Zhang X.O., Wu Z., Zhang S., Wang H.B., Ge J., Lu X. (2014). Human colorectal cancer-specific CCAT1-L lncRNA regulates long-range chromatin interactions at the MYC locus. Cell Res..

[54] Shen L., Wang Q., Liu R., Chen Z., Zhang X., Zhou P., Wang Z. (2018). LncRNA lnc-RI regulates homologous recombination repair of DNA double-strand breaks by stabilizing RAD51 mRNA as a competitive endogenous RNA. Nucleic Acids Res..

[55] Su Y., Zhang T., Tang J., Zhang L., Fan S., Zhou J., Liang C. (2021). Construction of Competitive Endogenous RNA Network and Verification of 3-Key LncRNA Signature Associated With Distant Metastasis and Poor Prognosis in Patients With Clear Cell Renal Cell Carcinoma. Front. Oncol..

[56] Gao N., Li Y., Li J., Gao Z., Yang Z., Li Y., Liu H., Fan T. (2020). Long Non-Coding RNAs: The Regulatory Mechanisms, Research Strategies, and Future Directions in Cancers. Front. Oncol..

[57] Zhang E., Han L., Yin D., He X., Hong L., Si X., Qiu M., Xu T., De W., Xu L. (2017). H3K27 acetylation activated-long non-coding RNA CCAT1 affects cell proliferation and migration by regulating SPRY4 and HOXB13 expression in esophageal squamous cell carcinoma. Nucleic Acids Res..

[58] Hadji F., Boulanger M.C., Guay S.P., Gaudreault N., Amellah S., Mkannez G., Bouchareb R., Marchand J.T., Nsaibia M.J., Guauque-Olarte S. (2016). Altered DNA Methylation of Long Noncoding RNA H19 in Calcific Aortic Valve Disease Promotes Mineralization by Silencing NOTCH1. Circulation.

[59] Xie J.J., Jiang Y.Y., Jiang Y., Li C.Q., Lim M.C., An O., Mayakonda A., Ding L.W., Long L., Sun C. (2018). Super-Enhancer-Driven Long Non-Coding RNA LINC01503, Regulated by TP63, Is Over-Expressed and Oncogenic in Squamous Cell Carcinoma. Gastroenterology.

[60] Hämmerle M., Gutschner T., Uckelmann H., Ozgur S., Fiskin E., Gross M., Skawran B., Geffers R., Longerich T., Breuhahn K. (2013). Posttranscriptional destabilization of the liver-specific long noncoding RNA HULC by the IGF2 mRNA-binding protein 1 (IGF2BP1). Hepatology.

[61] Jin C., Shi L., Li K., Liu W., Qiu Y., Zhao Y., Zhao B., Li Z., Li Y., Zhu Q. (2021). Mechanism of tumor-derived extracellular vesicles in regulating renal cell carcinoma progression by the delivery of MALAT1. Oncol. Rep..

[62] Watson D.C., Bayik D., Srivatsan A., Bergamaschi C., Valentin A., Niu G., Bear J., Monninger M., Sun M., Morales-Kastresana A. (2016). Efficient production and enhanced tumor delivery of engineered extracellular vesicles. Biomaterials.

[63] Ghoroghi S., Mary B., Asokan N., Goetz J.G., Hyenne V. (2021). Tumor extracellular vesicles drive metastasis (it’s a long way from home). FASEB Bioadv..

[64] Peinado H., Alečković M., Lavotshkin S., Matei I., Costa-Silva B., Moreno-Bueno G., Hergueta-Redondo M., Williams C., García-Santos G., Ghajar C. (2012). Melanoma exosomes educate bone marrow progenitor cells toward a pro-metastatic phenotype through MET. Nat. Med..

[65] Sabbagh Q., Andre-Gregoire G., Guevel L., Gavard J. (2020). Vesiclemia: Counting on extracellular vesicles for glioblastoma patients. Oncogene.

[66] Melo S.A., Luecke L.B., Kahlert C., Fernandez A.F., Gammon S.T., Kaye J., LeBleu V.S., Mittendorf E.A., Weitz J., Rahbari N. (2015). Glypican-1 identifies cancer exosomes and detects early pancreatic cancer. Nature.

[67] Mathieu M., Martin-Jaular L., Lavieu G., Théry C. (2019). Specificities of secretion and uptake of exosomes and other extracellular vesicles for cell-to-cell communication. Nat. Cell Biol..

[68] Yáñez-Mó M., Siljander P.R., Andreu Z., Zavec A.B., Borràs F.E., Buzas E.I., Buzas K., Casal E., Cappello F., Carvalho J. (2015). Biological properties of extracellular vesicles and their physiological functions. J. Extracell. Vesicles.

[69] Kalluri R., LeBleu V.S. (2020). The biology, function, and biomedical applications of exosomes. Science.

[70] Sheehan C., D’Souza-Schorey C. (2019). Tumor-derived extracellular vesicles: Molecular parcels that enable regulation of the immune response in cancer. J. Cell Sci..

[71] Marar C., Starich B., Wirtz D. (2021). Extracellular vesicles in immunomodulation and tumor progression. Nat. Immunol..

[72] Kosaka N., Iguchi H., Hagiwara K., Yoshioka Y., Takeshita F., Ochiya T. (2013). Neutral sphingomyelinase 2 (nSMase2)-dependent exosomal transfer of angiogenic microRNAs regulate cancer cell metastasis. J. Biol. Chem..

[73] Ghoroghi S., Mary B., Larnicol A., Asokan N., Klein A., Osmani N., Busnelli I., Delalande F., Paul N., Halary S. (2021). Ral GTPases promote breast cancer metastasis by controlling biogenesis and organ targeting of exosomes. Elife.

[74] Guo J., Duan Z., Zhang C., Wang W., He H., Liu Y., Wu P., Wang S., Song M., Chen H. (2020). Mouse 4T1 Breast Cancer Cell-Derived Exosomes Induce Proinflammatory Cytokine Production in Macrophages via miR-183. J. Immunol..

[75] Ma P., Pan Y., Li W., Sun C., Liu J., Xu T., Shu Y. (2017). Extracellular vesicles-mediated noncoding RNAs transfer in cancer. J. Hematol. Oncol..

[76] Xu R., Rai A., Chen M., Suwakulsiri W., Greening D.W., Simpson R.J. (2018). Extracellular vesicles in cancer-implications for future improvements in cancer care. Nat. Rev. Clin. Oncol..

[77] Zhao M., Wang S., Li Q., Ji Q., Guo P., Liu X. (2018). MALAT1: A long non-coding RNA highly associated with human cancers. Oncol. Lett..

[78] Ji P., Diederichs S., Wang W., Böing S., Metzger R., Schneider P.M., Tidow N., Brandt B., Buerger H., Bulk E. (2003). MALAT-1, a novel noncoding RNA, and thymosin beta4 predict metastasis and survival in early-stage non-small cell lung cancer. Oncogene.

[79] Guttman M., Amit I., Garber M., French C., Lin M.F., Feldser D., Huarte M., Zuk O., Carey B.W., Cassady J.P. (2009). Chromatin signature reveals over a thousand highly conserved large non-coding RNAs in mammals. Nature.

[80] Hirata H., Hinoda Y., Shahryari V., Deng G., Nakajima K., Tabatabai Z.L., Ishii N., Dahiya R. (2015). Long Noncoding RNA MALAT1 Promotes Aggressive Renal Cell Carcinoma through Ezh2 and Interacts with miR-205. Cancer Res..

[81] Wagener N., Holland D., Bulkescher J., Crnković-Mertens I., Hoppe-Seyler K., Zentgraf H., Pritsch M., Buse S., Pfitzenmaier J., Haferkamp A. (2008). The enhancer of zeste homolog 2 gene contributes to cell proliferation and apoptosis resistance in renal cell carcinoma cells. Int. J. Cancer.

[82] Chen S., Ma P., Zhao Y., Li B., Jiang S., Xiong H., Wang Z., Wang H., Jin X., Liu C. (2017). Biological function and mechanism of MALAT-1 in renal cell carcinoma proliferation and apoptosis: Role of the MALAT-1-Livin protein interaction. J. Physiol. Sci..

[83] Zhai W., Ma J., Zhu R., Xu C., Zhang J., Chen Y., Chen Z., Gong D., Zheng J., Chen C. (2018). MiR-532-5p suppresses renal cancer cell proliferation by disrupting the ETS1-mediated positive feedback loop with the KRAS-NAP1L1/P-ERK axis. Br. J. Cancer.

[84] Luo Y., Liu F., Yan C., Qu W., Zhu L., Guo Z., Zhou F., Zhang W. (2020). Long Non-Coding RNA CASC19 Sponges microRNA-532 and Promotes Oncogenicity of Clear Cell Renal Cell Carcinoma by Increasing ETS1 Expression. Cancer Manag. Res..

[85] Qiu J.J., Lin X.J., Tang X.Y., Zheng T.T., Lin Y.Y., Hua K.Q. (2018). Exosomal Metastasis-Associated Lung Adenocarcinoma Transcript 1 Promotes Angiogenesis and Predicts Poor Prognosis in Epithelial Ovarian Cancer. Int. J. Biol. Sci..

[86] Hardin H., Helein H., Meyer K., Robertson S., Zhang R., Zhong W., Lloyd R.V. (2018). Thyroid cancer stem-like cell exosomes: Regulation of EMT via transfer of lncRNAs. Lab. Investig..

[87] Li Z., Ma Z., Xu X. (2019). Long non-coding RNA MALAT1 correlates with cell viability and mobility by targeting miR-22-3p in renal cell carcinoma via the PI3K/Akt pathway. Oncol. Rep..

[88] Syn N., Wang L., Sethi G., Thiery J.P., Goh B.C. (2016). Exosome-Mediated Metastasis: From Epithelial-Mesenchymal Transition to Escape from Immunosurveillance. Trends Pharm. Sci..

[89] Zhang Y., Guan X., Wang H., Wang Y., Yue D., Chen R. (2020). Long non-coding RNA metastasis-associated lung adenocarcinoma transcript 1 regulates renal cancer cell migration via cofilin-1. Oncol. Lett..

[90] Tripathi V., Ellis J.D., Shen Z., Song D.Y., Pan Q., Watt A.T., Freier S.M., Bennett C.F., Sharma A., Bubulya P.A. (2010). The nuclear-retained noncoding RNA MALAT1 regulates alternative splicing by modulating SR splicing factor phosphorylation. Mol. Cell.

[91] Chen R., Liu Y., Zhuang H., Yang B., Hei K., Xiao M., Hou C., Gao H., Zhang X., Jia C. (2017). Quantitative proteomics reveals that long non-coding RNA MALAT1 interacts with DBC1 to regulate p53 acetylation. Nucleic Acids Res..

[92] Engreitz J.M., Sirokman K., McDonel P., Shishkin A.A., Surka C., Russell P., Grossman S.R., Chow A.Y., Guttman M., Lander E.S. (2014). RNA-RNA interactions enable specific targeting of noncoding RNAs to nascent Pre-mRNAs and chromatin sites. Cell.

[93] Bravo-Cordero J.J., Magalhaes M.A., Eddy R.J., Hodgson L., Condeelis J. (2013). Functions of cofilin in cell locomotion and invasion. Nat. Rev. Mol. Cell Biol..

[94] Wang W., Eddy R., Condeelis J. (2007). The cofilin pathway in breast cancer invasion and metastasis. Nat. Rev. Cancer.

[95] Guo R., Zou B., Liang Y., Bian J., Xu J., Zhou Q., Zhang C., Chen T., Yang M., Wang H. (2021). LncRNA RCAT1 promotes tumor progression and metastasis via miR-214-5p/E2F2 axis in renal cell carcinoma. Cell Death Dis..

[96] Chen Y.R., Wu Y.S., Wang W.S., Zhang J.S., Wu Q.G. (2020). Upregulation of lncRNA DANCR functions as an oncogenic role in non-small lung cancer by regulating miR-214-5p/CIZ1 axis. Eur. Rev. Med. Pharm. Sci..

[97] Guo M., Lin B., Li G., Lin J., Jiang X. (2020). LncRNA TDRG1 promotes the proliferation, migration, and invasion of cervical cancer cells by sponging miR-214-5p to target SOX4. J. Recept. Signal Transduct. Res..

[98] Apostolou A., Poreau B., Delrieu L., Thévenon J., Jouk P.S., Lallemand G., Emadali A., Sartelet H. (2020). High Activation of the AKT Pathway in Human Multicystic Renal Dysplasia. Pathobiology.

[99] Xu H., Xu W.-H., Ren F., Wang J., Wang H.-K., Cao D.-L., Shi G.-H., Qu Y.-Y., Zhang H.-L., Ye D.-W. (2020). Prognostic value of epithelial-mesenchymal transition markers in clear cell renal cell carcinoma. Aging.

[100] Jonasch E., Gao J., Rathmell W.K. (2014). Renal cell carcinoma. BMJ.

[101] Vaidya A.M., Sun Z., Ayat N., Schilb A., Liu X., Jiang H., Sun D., Scheidt J., Qian V., He S. (2019). Systemic Delivery of Tumor-Targeting siRNA Nanoparticles against an Oncogenic LncRNA Facilitates Effective Triple-Negative Breast Cancer Therapy. Bioconjug. Chem..

[102] Tan L., Tang Y., Li H., Li P., Ye Y., Cen J., Gui C., Luo J., Cao J., Wei J. (2021). N6-Methyladenosine Modification of LncRNA DUXAP9 Promotes Renal Cancer Cells Proliferation and Motility by Activating the PI3K/AKT Signaling Pathway. Front. Oncol..

[103] Xu M., Xu L., Wang Y., Dai G., Xue B., Liu Y.Y., Zhu J., Zhu J. (2020). BRD4 inhibition sensitizes renal cell carcinoma cells to the PI3K/mTOR dual inhibitor VS-5584. Aging.

[104] Merseburger A.S., Hennenlotter J., Kuehs U., Simon P., Kruck S., Koch E., Stenzl A., Kuczyk M.A. (2008). Activation of PI3K is associated with reduced survival in renal cell carcinoma. Urol. Int..

[105] Creighton C.J., Morgan M., Gunaratne P.H., Wheeler D.A., Gibbs R.A., Gordon Robertson A., Chu A., Beroukhim R., Cibulskis K., Signoretti S. (2013). Comprehensive molecular characterization of clear cell renal cell carcinoma. Nature.

[106] Erin N., Grahovac J., Brozovic A., Efferth T. (2020). Tumor microenvironment and epithelial mesenchymal transition as targets to overcome tumor multidrug resistance. Drug Resist. Updates.

[107] Zhu T., An S., Choy M.T., Zhou J., Wu S., Liu S., Liu B., Yao Z., Zhu X., Wu J. (2019). LncRNA DUXAP9-206 directly binds with Cbl-b to augment EGFR signaling and promotes non-small cell lung cancer progression. J. Cell Mol. Med..

[108] Wang Z.L., Wang C., Liu W., Ai Z.L. (2019). Emerging roles of the long non-coding RNA 01296/microRNA-143-3p/MSI2 axis in development of thyroid cancer. Biosci. Rep..

[109] Chen J., Lou W., Ding B., Wang X. (2019). Overexpressed pseudogenes, DUXAP8 and DUXAP9, promote growth of renal cell carcinoma and serve as unfavorable prognostic biomarkers. Aging.

[110] Kulkarni P., Dasgupta P., Hashimoto Y., Shiina M., Shahryari V., Tabatabai Z.L., Yamamura S., Tanaka Y., Saini S., Dahiya R. (2021). A lncRNA TCL6-miR-155 Interaction Regulates the Src-Akt-EMT Network to Mediate Kidney Cancer Progression and Metastasis. Cancer Res..

[111] Yang K., Lu X.F., Luo P.C., Zhang J. (2018). Identification of Six Potentially Long Noncoding RNAs as Biomarkers Involved Competitive Endogenous RNA in Clear Cell Renal Cell Carcinoma. Biomed. Res. Int..

[112] Wang J., Zhang C., He W., Gou X. (2018). Construction and comprehensive analysis of dysregulated long non-coding RNA-associated competing endogenous RNA network in clear cell renal cell carcinoma. J. Cell Biochem..

[113] Liu L.P., Gong Y.B. (2018). LncRNA-TCL6 promotes early abortion and inhibits placenta implantation via the EGFR pathway. Eur. Rev. Med. Pharm. Sci..

[114] Roelants C., Giacosa S., Pillet C., Bussat R., Champelovier P., Bastien O., Guyon L., Arnoux V., Cochet C., Filhol O. (2018). Combined inhibition of PI3K and Src kinases demonstrates synergistic therapeutic efficacy in clear-cell renal carcinoma. Oncotarget.

[115] Yonezawa Y., Nagashima Y., Sato H., Virgona N., Fukumoto K., Shirai S., Hagiwara H., Seki T., Ariga T., Senba H. (2005). Contribution of the Src family of kinases to the appearance of malignant phenotypes in renal cancer cells. Mol. Carcinog..

[116] Su H., Sun T., Wang H., Shi G., Zhang H., Sun F., Ye D. (2017). Decreased TCL6 expression is associated with poor prognosis in patients with clear cell renal cell carcinoma. Oncotarget.

[117] Yang F.Y., Wang Y., Wu J.G., Song S.L., Huang G., Xi W.M., Tan L.L., Wang J., Cao Q. (2017). Analysis of long non-coding RNA expression profiles in clear cell renal cell carcinoma. Oncol. Lett..

[118] Shen P., Qu L., Wang J., Ding Q., Zhou C., Xie R., Wang H., Ji G. (2021). LncRNA LINC00342 contributes to the growth and metastasis of colorectal cancer via targeting miR-19a-3p/NPEPL1 axis. Cancer Cell Int..

[119] Dias A.S., Almeida C.R., Helguero L.A., Duarte I.F. (2019). Metabolic crosstalk in the breast cancer microenvironment. Eur. J. Cancer.

[120] Nenkov M., Ma Y., Gaßler N., Chen Y. (2021). Metabolic Reprogramming of Colorectal Cancer Cells and the Microenvironment: Implication for Therapy. Int. J. Mol. Sci..

[121] Wu M., Fu P., Qu L., Liu J., Lin A. (2020). Long Noncoding RNAs, New Critical Regulators in Cancer Immunity. Front. Oncol..

[122] Cai C.F., Ye G.D., Shen D.Y., Zhang W., Chen M.L., Chen X.X., Han D.X., Mi Y.J., Luo Q.C., Cai W.Y. (2018). Chibby suppresses aerobic glycolysis and proliferation of nasopharyngeal carcinoma via the Wnt/β-catenin-Lin28/let7-PDK1 cascade. J. Exp. Clin. Cancer Res..

[123] Jiang Y., Han Q., Zhao H., Zhang J. (2021). Promotion of epithelial-mesenchymal transformation by hepatocellular carcinoma-educated macrophages through Wnt2b/β-catenin/c-Myc signaling and reprogramming glycolysis. J. Exp. Clin. Cancer Res..

[124] Lee S.Y., Jeon H.M., Ju M.K., Kim C.H., Yoon G., Han S.I., Park H.G., Kang H.S. (2012). Wnt/Snail signaling regulates cytochrome C oxidase and glucose metabolism. Cancer Res..

[125] Gao L., Zhao A., Wang X. (2020). Upregulation of lncRNA AGAP2-AS1 is an independent predictor of poor survival in patients with clear cell renal carcinoma. Oncol. Lett..

[126] Fendler A., Stephan C., Yousef G.M., Kristiansen G., Jung K. (2016). The translational potential of microRNAs as biofluid markers of urological tumours. Nat. Rev. Urol..

[127] Tian Y., Zheng Y., Dong X. (2019). AGAP2-AS1 serves as an oncogenic lncRNA and prognostic biomarker in glioblastoma multiforme. J. Cell Biochem..

[128] Fan K.J., Liu Y., Yang B., Tian X.D., Li C.R., Wang B. (2017). Prognostic and diagnostic significance of long non-coding RNA AGAP2-AS1 levels in patients with non-small cell lung cancer. Eur. Rev. Med. Pharm. Sci..

[129] Luo W., Li X., Song Z., Zhu X., Zhao S. (2019). Long non-coding RNA AGAP2-AS1 exerts oncogenic properties in glioblastoma by epigenetically silencing TFPI2 through EZH2 and LSD1. Aging.

[130] Hui B., Ji H., Xu Y., Wang J., Ma Z., Zhang C., Wang K., Zhou Y. (2019). RREB1-induced upregulation of the lncRNA AGAP2-AS1 regulates the proliferation and migration of pancreatic cancer partly through suppressing ANKRD1 and ANGPTL4. Cell Death Dis..

[131] Liu Z., Wang Y., Wang L., Yao B., Sun L., Liu R., Chen T., Niu Y., Tu K., Liu Q. (2019). Long non-coding RNA AGAP2-AS1, functioning as a competitive endogenous RNA, upregulates ANXA11 expression by sponging miR-16-5p and promotes proliferation and metastasis in hepatocellular carcinoma. J. Exp. Clin. Cancer Res..

[132] Wang W., Yang F., Zhang L., Chen J., Zhao Z., Wang H., Wu F., Liang T., Yan X., Li J. (2016). LncRNA profile study reveals four-lncRNA signature associated with the prognosis of patients with anaplastic gliomas. Oncotarget.

[133] Zheng Z., Chen M., Xing P., Yan X., Xie B. (2019). Increased Expression of Exosomal AGAP2-AS1 (AGAP2 Antisense RNA 1) In Breast Cancer Cells Inhibits Trastuzumab-Induced Cell Cytotoxicity. Med. Sci. Monit..

[134] Varambally S., Yu J., Laxman B., Rhodes D.R., Mehra R., Tomlins S.A., Shah R.B., Chandran U., Monzon F.A., Becich M.J. (2005). Integrative genomic and proteomic analysis of prostate cancer reveals signatures of metastatic progression. Cancer Cell.

[135] Zheng Y., Lu S., Xu Y., Zheng J. (2019). Long non-coding RNA AGAP2-AS1 promotes the proliferation of glioma cells by sponging miR-15a/b-5p to upregulate the expression of HDGF and activating Wnt/β-catenin signaling pathway. Int. J. Biol. Macromol..

[136] Ramjiawan R.R., Griffioen A.W., Duda D.G. (2017). Anti-angiogenesis for cancer revisited: Is there a role for combinations with immunotherapy?. Angiogenesis.

[137] Chen T., You Y., Jiang H., Wang Z.Z. (2017). Epithelial-mesenchymal transition (EMT): A biological process in the development, stem cell differentiation, and tumorigenesis. J. Cell Physiol..

[138] Braune E.B., Lendahl U. (2016). Notch -- a goldilocks signaling pathway in disease and cancer therapy. Discov. Med..

[139] Lugano R., Ramachandran M., Dimberg A. (2020). Tumor angiogenesis: Causes, consequences, challenges and opportunities. Cell Mol. Life Sci..

[140] Chappell J.C., Payne L.B., Rathmell W.K. (2019). Hypoxia, angiogenesis, and metabolism in the hereditary kidney cancers. J. Clin. Investig..

[141] Fu S., Gong B., Wang S., Chen Q., Liu Y., Zhuang C., Li Z., Zhang Z., Ma M., Sun T. (2021). Prognostic Value of Long Noncoding RNA DLEU2 and Its Relationship with Immune Infiltration in Kidney Renal Clear Cell Carcinoma and Liver Hepatocellular Carcinoma. Int. J. Gen. Med..

[142] Xu W., Wang B., Cai Y., Guo C., Liu K., Yuan C. (2021). DLEU2: A Meaningful Long Noncoding RNA in Oncogenesis. Curr. Pharm. Des..

[143] Mian M., Scandurra M., Chigrinova E., Shen Y., Inghirami G., Greiner T.C., Chan W.C., Vose J.M., Testoni M., Chiappella A. (2012). Clinical and molecular characterization of diffuse large B-cell lymphomas with 13q14.3 deletion. Ann. Oncol..

[144] Han S., Qi Y., Xu Y., Wang M., Wang J., Wang J., Yuan M., Jia Y., Ma X., Wang Y. (2021). lncRNA DLEU2 promotes gastric cancer progression through ETS2 via targeting miR-30a-5p. Cancer Cell Int..

[145] He M., Wang Y., Cai J., Xie Y., Tao C., Jiang Y., Li H., Song F. (2021). LncRNA DLEU2 promotes cervical cancer cell proliferation by regulating cell cycle and NOTCH pathway. Exp. Cell Res..

[146] Li G., Zhang Z., Chen Z., Liu B., Wu H. (2021). LncRNA DLEU2 is activated by STAT1 and induces gastric cancer development via targeting miR-23b-3p/NOTCH2 axis and Notch signaling pathway. Life Sci..

[147] Bao S., Jiang X., Jin S., Tu P., Lu J. (2021). TGF-β1 induces immune escape by enhancing PD-1 and CTLA-4 expression on T lymphocytes in hepatocellular carcinoma. Front. Oncol..

[148] Poli A., Abdul-Hamid S., Zaurito A.E., Campagnoli F., Bevilacqua V., Sheth B., Fiume R., Pagani M., Abrignani S., Divecha N. (2021). PIP4Ks impact on PI3K, FOXP3, and UHRF1 signaling and modulate human regulatory T cell proliferation and immunosuppressive activity. Proc. Natl. Acad. Sci. USA.

[149] Sawant A., Hensel J.A., Chanda D., Harris B.A., Siegal G.P., Maheshwari A., Ponnazhagan S. (2012). Depletion of plasmacytoid dendritic cells inhibits tumor growth and prevents bone metastasis of breast cancer cells. J. Immunol..

[150] Xing Z., Zhang M., Liu J., Liu G., Feng K., Wang X. (2021). LINC00337 induces tumor development and chemoresistance to paclitaxel of breast cancer by recruiting M2 tumor-associated macrophages. Mol. Immunol..

[151] Klein U., Lia M., Crespo M., Siegel R., Shen Q., Mo T., Ambesi-Impiombato A., Califano A., Migliazza A., Bhagat G. (2010). The DLEU2/miR-15a/16-1 cluster controls B cell proliferation and its deletion leads to chronic lymphocytic leukemia. Cancer Cell.

[152] Dong P., Xiong Y., Konno Y., Ihira K., Kobayashi N., Yue J., Watari H. (2021). Long non-coding RNA DLEU2 drives EMT and glycolysis in endometrial cancer through HK2 by competitively binding with miR-455 and by modulating the EZH2/miR-181a pathway. J. Exp. Clin. Cancer Res..

[153] Ma J., Qi G., Li L. (2020). LncRNA NNT-AS1 promotes lung squamous cell carcinoma progression by regulating the miR-22/FOXM1 axis. Cell. Mol. Biol. Lett..

[154] Liu Y., Guo R., Qiao Y., Han L., Liu M. (2020). LncRNA NNT-AS1 contributes to the cisplatin resistance of cervical cancer through NNT-AS1/miR-186/HMGB1 axis. Cancer Cell Int..

[155] Winkle M., El-Daly S.M., Fabbri M., Calin G.A. (2021). Noncoding RNA therapeutics—Challenges and potential solutions. Nat. Rev. Drug Discov..

[156] Zhang Y., Wang Y. (2021). Circular RNAs in Hepatocellular Carcinoma: Emerging Functions to Clinical Significances. Front. Oncol..

[157] Li K., Sun D., Gou Q., Ke X., Gong Y., Zuo Y., Zhou J.K., Guo C., Xia Z., Liu L. (2018). Long non-coding RNA linc00460 promotes epithelial-mesenchymal transition and cell migration in lung cancer cells. Cancer Lett..

[158] Yue Q.Y., Zhang Y. (2018). Effects of Linc00460 on cell migration and invasion through regulating epithelial-mesenchymal transition (EMT) in non-small cell lung cancer. Eur. Rev. Med. Pharm. Sci..

[159] Meng X., Sun W., Yu J., Zhou Y., Gu Y., Han J., Zhou L., Jiang X., Wang C. (2020). LINC00460-miR-149-5p/miR-150-5p-Mutant p53 Feedback Loop Promotes Oxaliplatin Resistance in Colorectal Cancer. Mol. Nucleic Acids.

[160] Zhang D., Zeng S., Hu X. (2020). Identification of a three-long noncoding RNA prognostic model involved competitive endogenous RNA in kidney renal clear cell carcinoma. Cancer Cell Int..

[161] Yuan B., Yang J., Gu H., Ma C. (2020). Down-regulation of LINC00460 represses metastasis of colorectal cancer via WWC2. Dig. Dis. Sci..

[162] Zhang S., Xu J., Wang H., Guo H. (2019). Downregulation of long noncoding RNA LINC00460 expression suppresses tumor growth in vitro and in vivo in gastric cancer. Cancer Biomark..

[163] Wierstra I. (2013). FOXM1 (Forkhead box M1) in tumorigenesis: Overexpression in human cancer, implication in tumorigenesis, oncogenic functions, tumor-suppressive properties, and target of anticancer therapy. Adv. Cancer Res..

[164] Jin L., Li Y., Liu J., Yang S., Gui Y., Mao X., Nie G., Lai Y. (2016). Tumor suppressor miR-149-5p is associated with cellular migration, proliferation and apoptosis in renal cell carcinoma. Mol. Med. Rep..

[165] Xie M., Lv Y., Liu Z., Zhang J., Liang C., Liao X., Liang R., Lin Y., Li Y. (2018). Identification and validation of a four-miRNA (miRNA-21-5p, miRNA-9-5p, miR-149-5p, and miRNA-30b-5p) prognosis signature in clear cell renal cell carcinoma. Cancer Manag. Res..

[166] Joung J., Engreitz J.M., Konermann S., Abudayyeh O.O., Verdine V.K., Aguet F., Gootenberg J.S., Sanjana N.E., Wright J.B., Fulco C.P. (2017). Genome-scale activation screen identifies a lncRNA locus regulating a gene neighbourhood. Nature.

[167] Bellucci M., Agostini F., Masin M., Tartaglia G.G. (2011). Predicting protein associations with long noncoding RNAs. Nat. Methods.

[168] Mizutani A., Koinuma D., Seimiya H., Miyazono K. (2016). The Arkadia-ESRP2 axis suppresses tumor progression: Analyses in clear-cell renal cell carcinoma. Oncogene.

